# DNA barcoding, multilocus phylogeny, and morphometry reveal phenotypic plasticity in the Chinese freshwater mussel *Lamprotula caveata* (Bivalvia: Unionidae)

**DOI:** 10.1002/ece3.9035

**Published:** 2022-07-13

**Authors:** Ruiwen Wu, Xiongjun Liu, Liang Guo, Chunhua Zhou, Shan Ouyang, Xiaoping Wu

**Affiliations:** ^1^ School of Life Science Shanxi Normal University Taiyuan China; ^2^ School of Life Science Jiaying University Meizhou China; ^3^ Fuzhou Wilds of Insects Cultural Creativity Co., Ltd. Fuzhou China; ^4^ School of Life Sciences Nanchang University Nanchang China

**Keywords:** China, DNA barcode, *Lamprotula*, molecular clock, morphometrics, phenotypic plasticity

## Abstract

Accurate species identification is crucial for developing conservation strategies for freshwater mussels, one of the most imperiled faunas in the world. Traditionally, mussel species description primarily relied on conchological characters. However, shell morphology has great variability, which leads to the complexity of species delimitation. As endemic species to China, *Lamprotula caveata* was originally described by Heude (1877). *Lamprotula quadrangulosus* and *Lamprotula contritus* were considered for synonymization of *L. caveata* based on shell variants in the early 20th century, which has been long debated due to lack of rigorous molecular analysis. Moreover, great morphological variation caused doubt whether there are cryptic species. In this study, we used a combined phylogenetic and morphometric approach to verify the validity of the synonymization of *L. caveata*. The results of molecular species delimitation showed that two molecular operational taxonomic units (MOTUs) were identified in *Lamprotula* spp., including the *L. leaii* lineage and the complex lineage (*L. quadrangulosa*, *L. cornuumlunae*, *L. contritus*, and *L. caveata*). Phylogenetic analyses revealed that *L. cornuumlunae* formed a basal monophyletic clade, whose divergence time was relatively recent (4.26 Ma [95% HPD = 1.91–7.22 Ma]), and *L. contritus*, *L. caveata*, and *L. quadrangulosa* formed a large polytomy group with very shallow branches. In the previous study, we have demonstrated the validity of *L. cornuumlunae*. The molecular evidences supported that the complex (*L. quadrangulosa* + *L. contritus* + *L. caveata*) was a valid species; *L. quadrangulosa* and *L. contritus* were synonyms of *L. caveata*. In addition, three morphospecies (*L. quadrangulosa*, *L. contritus*, and *L. caveata*) were aggregated without clear differentiation based on shell morphometric analysis. We confirmed multiple phenotypes in *L. caveata* for species identification and presumed that the phenotypic plasticity was a response to specific habitats. This study clarified the diversity and phylogeny of the *Lamprotula* group, which is a crucial step for developing new conservation and management strategies for this imperiled group.

## Graphical abstract

Using DNA barcoding, six‐molecule markers and morphometry, we supported that L. quadrangulosa and L. contritus were synonyms of L. caveata, and confirmed multiple phenotypes in L. caveata for species identification.

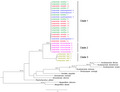

## 1 INTRODUCTION

Taxonomic uncertainties can seriously hinder the conservation of endangered species because inaccuracy in the species delineation may lead to incorrect estimates of biodiversity and flawed management decisions (Frankham, 2010; Geist & Kuehn, 2005; Isaac et al., 2004). Freshwater mussels (order Unionoida) are one of the most threatened animal groups in the world (Lydeard et al., 2004). Traditionally, freshwater malacologists primarily relied on conchological characters (e.g., shell shape, size, and color) for mussel species identifications (Haas, 1969; Heude, 1875–1885; Simpson, 1900, 1914). However, mollusks are heavily influenced by environmental conditions and their overall form exhibits considerable variability in shell morphology (Inoue et al., 2013; Zieritz et al., 2010, 2012), leading to the complexity of species delimitation.

*Lamprotula caveata* (Heude, 1877) is endemic to China (Graf & Cummings, 2021; Hu, 2005; Zieritz et al., 2017) and was initially described by Pierre Marie Heude (1877) who described Chinese unionid taxa by recognizing subtle shell variants. Species description based on shell morphology leads to an overestimation of species diversity due to morphological variability. There are still fewer studies applying genetic techniques for the characterization of species presences and identities in Asian freshwater systems compared with Europe or North America, despite this area being a biodiversity hotspot (Belle et al., 2019). Simpson (1914) believed that *Lamprotula* (*Unio*) *contritus* (Heude, 1881) and *Lamprotula* (*Unio*) *quadrangulosus* (Heude, 1881) were not morphologically distinct from *L. caveata* and recognized *L. quadrangulosus* and *L. contritus* as the synonym of *L. caveata*. Chinese malacologist Lin (1962), by examining Heude's holotype specimens, asserted that the characters of these three species were different in morphology and still accepted Heude's classification. Later revisions for synonymization have been long debated due to lack of rigorous molecular analysis (Haas, 1969; Liu et al., 1979).

Due to scarce sequence data for phylogenetic studies, we subsequently conducted molecular analyses on the above *Lamprotula* taxa. After BLAST searches on COI, lists of BLAST Hits were generated showing sequence homology to *L. caveata*. Accordingly, we proposed the hypothesis that *L. quadrangulosus* and *L. contritus* were the synonym of *L. caveata*.

*Lamprotula caveata* also has great variations in shell morphology based on our sampling experience. The identification of *L. caveata* is based on the conchological diagnosis characteristics that the shell surface is rough and uneven, and the concave and convex positions of the left and right shells correspond to each other (Liu et al., 1979). But, the great morphological variation caused doubt whether there are cryptic species in *L. caveata*.

Using the molecular phylogenetic methods to define species is becoming more prevalent, especially the application and development of DNA barcoding and multilocus molecular data, further encouraging the species definition and new species discovery (Araujo et al., 2018; Bolotov, Vikhrev, et al., 2017; Smith et al., 2019). In this study, we collected *Lamprotula quadrangulosus*, *Lamprotula contritus*, and *Lamprotula caveata* with different variations in shell morphology from the Poyang Lake Basin and also collected other *Lamprotula* taxa (e.g., *L. leaii*, *L. cornuumlunae*). Using DNA barcoding, six‐gene markers (the mitochondrial 16S rRNA, cytochrome c oxidase subunit I (COI) and NADH dehydrogenase subunit 1 (ND1), and the nuclear 18S rRNA, 28S rRNA, and histone H3) and shell morphometry, we implemented two purposes: (1) verifying the synonymization of *L. caveata*; (2) examining whether there were cryptic species in various morphology of *L. caveata*.

## 2 MATERIALS AND METHODS

### 2.1 Specimen collection

**FIGURE 1:**
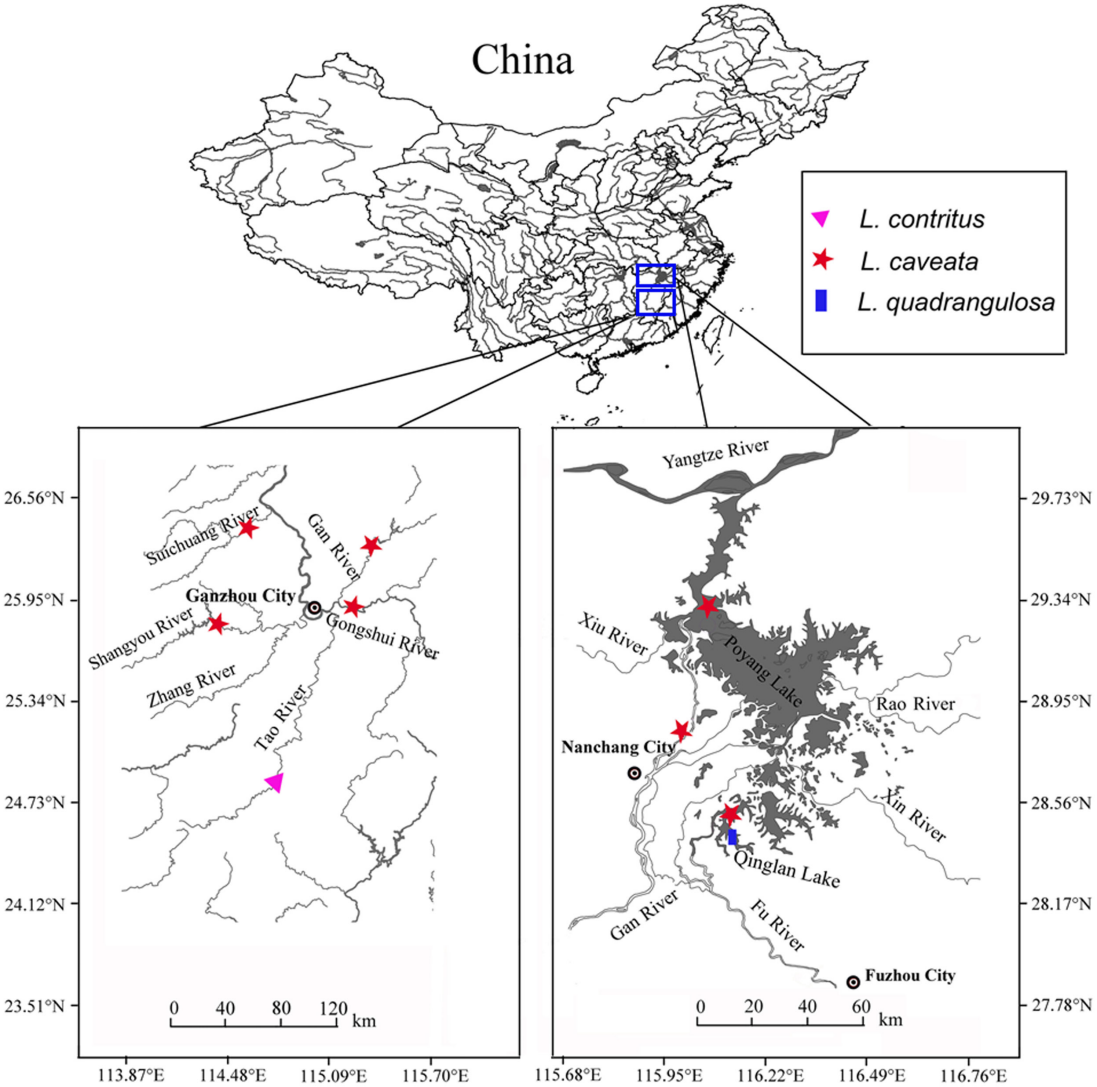
Sampling map of the *Lamprotula* species in this study. Red five‐pointed star indicates *L. caveata* sampling site; blue rectangle indicates *L. quadrangulosa* sampling site; triangle indicates *L. contritus* sampling site

In 2019–2021, 94 samples of *Lamprotula caveata*, *Lamprotula quadrangulosus*, and *Lamprotula contritus* were collected from the Gan River, Tao River, Fu River, Suichuang River, Shangyou River, Gongshui River, Qinlan Lake and Poyang Lake, Jiangxi Province, China (Figure 1). We also collected other *Lamprotula* species, that is, *Lamprotula leaii* and *Lamprotula cornuumlunae* to increase phylogenetic resolution. Morphospecies identification based on conchological characteristics in published literatures (Table 1; Heude, 1877; Liu et al., 1979; He & Zhuang, 2013) and the MUSSEL Project Web Site (http://musselproject.uwsp.edu/fmuotwaolcb/validsp_2816_syn.html). All specimens were deposited in the Biological Museum of Nanchang University.

**TABLE 1:** Conchological characteristics of *Lamprotula* morphological species. Numbers of shell length, width, and height are mean values with ranges

	*L. caveata*	*L. Quadrangulosus*	*L. Contritus*
Shell length (mm)	69.8 (43.6–90.3)	70.7 (58.8–82.9)	38.2 (25.9–47.5)
Shell width (mm)	30.4 (21.5–40.6)	28.5 (20.9–34.5)	15.4 (11.4–18.6)
Shell height (mm)	45.7 (31.5–62.8)	41.5 (28.0–53.8)	24.1 (17.3–27.5)
Beak cavity	Deep	Deep	Shallow
Umbo position	Front of back edge	Front of back edge	1/3 of back edge
Shell thickness	Thick	Thick	Thin‐medium
Pseudocardinal teeth	Thick and big	Thick and big	Thin and small
Pseudocardinal teeth sculpture	Yes	Yes	Yes
Lateral teeth	Well development	Well development	Reduce
Lateral teeth sculpture	Yes	Yes	Yes
Nacre color	White	White	White peach umbo area
Surface sculpture	irregular; nodules variable in quantity	Even; without nodules	Even; few of nodules
Posterior adductor muscle	Smooth	Smooth	Smooth
Anterior adductor muscle	Rough	Rough	Rough

**FIGURE 2:**
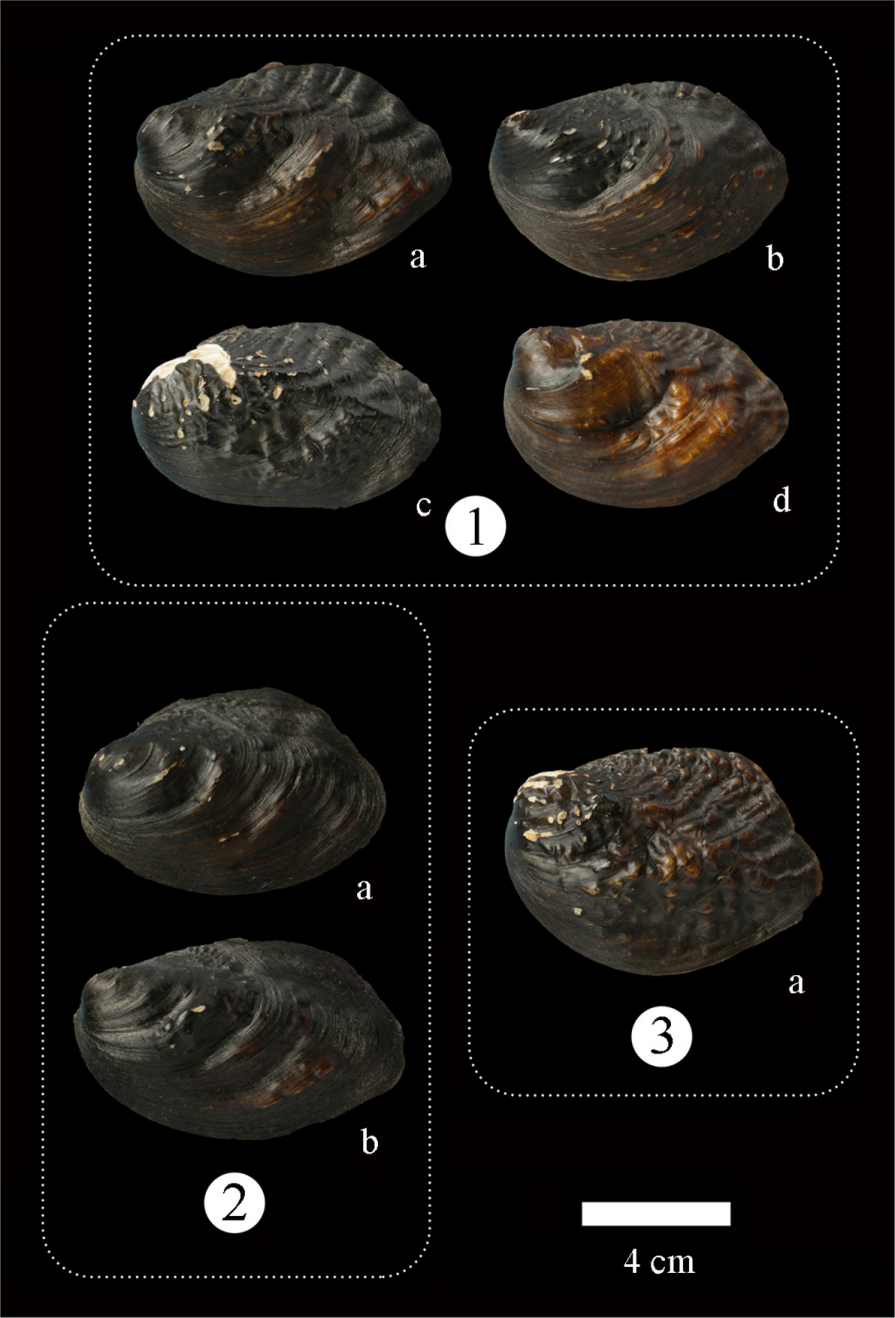
Variable shell morphology of *Lamprotula caveata*. The Arabic numerals in the figure indicate the three variability in shell phenotype. Letters denote specimens used for molecular data. The specimen numbers in the figure correspond to those in Table S1, Figures 5 and 6

**FIGURE 3:**
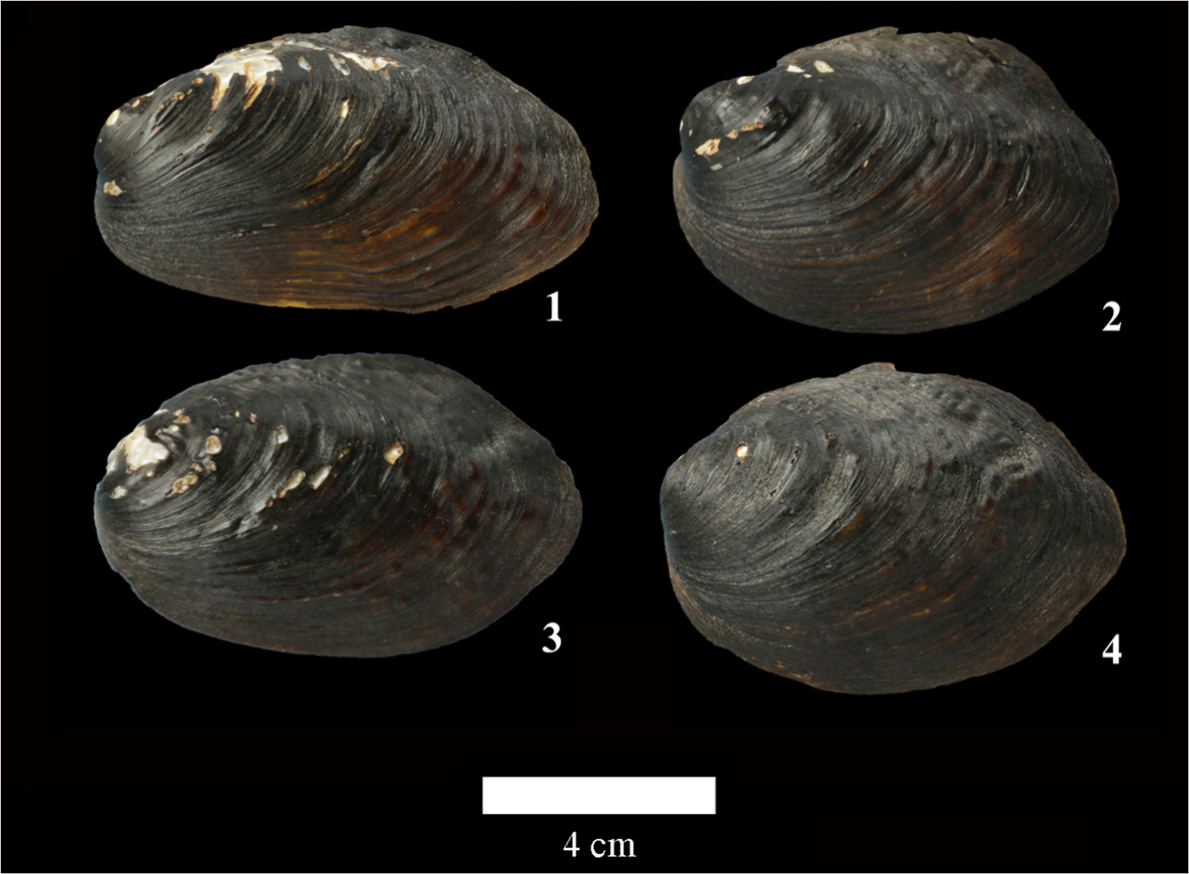
Morphospecies *Lamprotula quadrangulosa* shell morphology. Arabic numerals in the figure denote specimens used for molecular data. The specimen numbers correspond to those in Table S1, Figures 5 and 6

**FIGURE 4:**
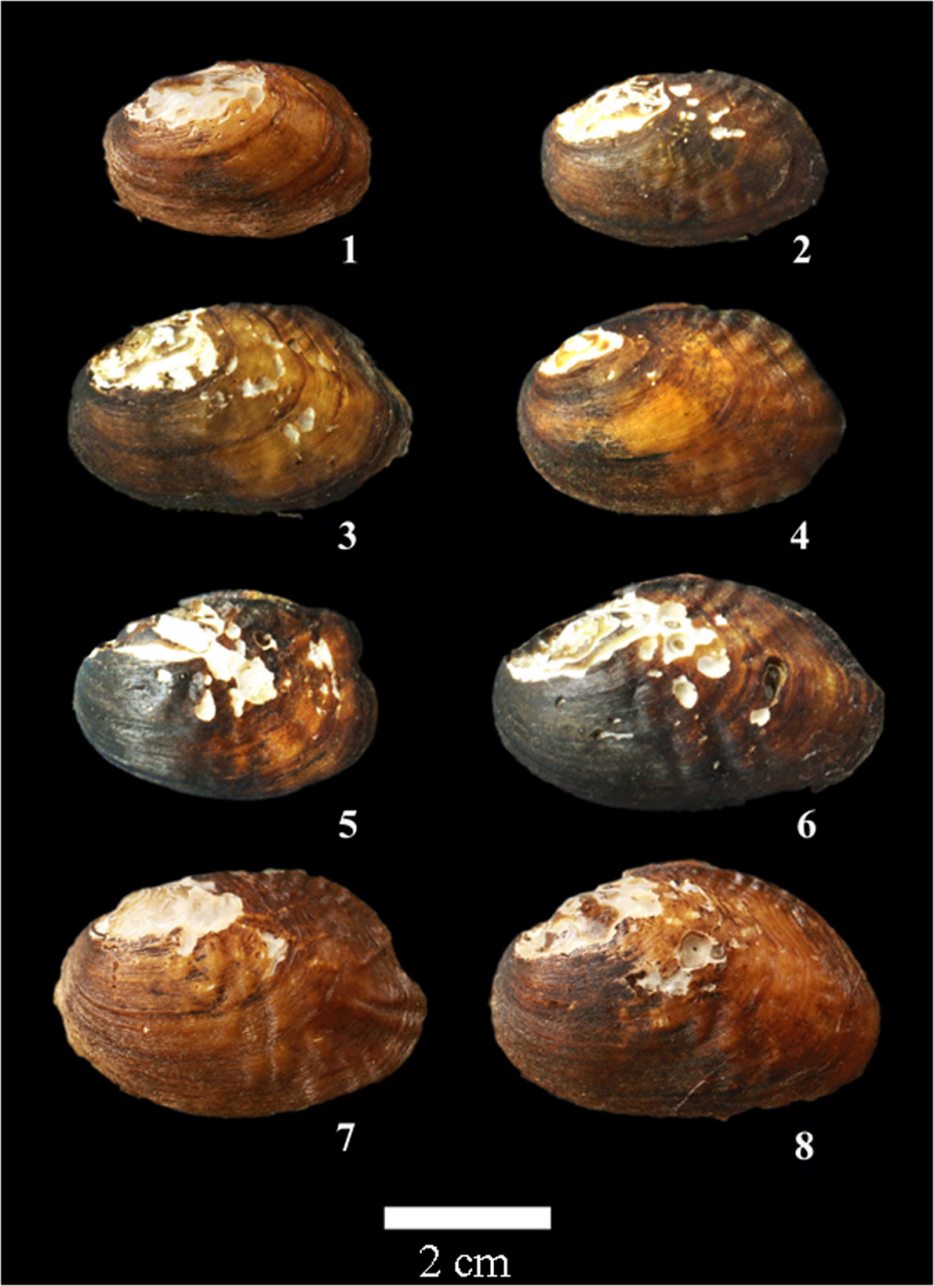
Morphospecies *Lamprotula contritus* shell morphology. Arabic numerals in the figure denote specimens used for molecular data. The specimen numbers correspond to those in Table S1, Figures 5 and 6

In all *Lamprotula caveata* specimens, we selected seven *Lamprotula caveata* specimens representing the variability in shell phenotype and categorized them into three main groups (Figure 2). 1 type: strong ridges and having a few nodules on the central of shell (Figure 2‐1); 2 type: no strong ridges and having few nodules on the surface (Figure 2‐2); 3 type: strong ridges and full of nodules on the surface (Figure 2‐3). As a result, seven *L. caveata*, four *L. quadrangulosa* (Figure 3), eight *Lamprotula contritus* (Figure 4), five *L. cornuumlunae*, and four *L. leaii* were used for the following molecular analysis. The collection information of *Lamprotula* species is shown in Table S1.

### 2.2 DNA extraction and mitochondrial DNA barcoding sequencing

**FIGURE 5:**
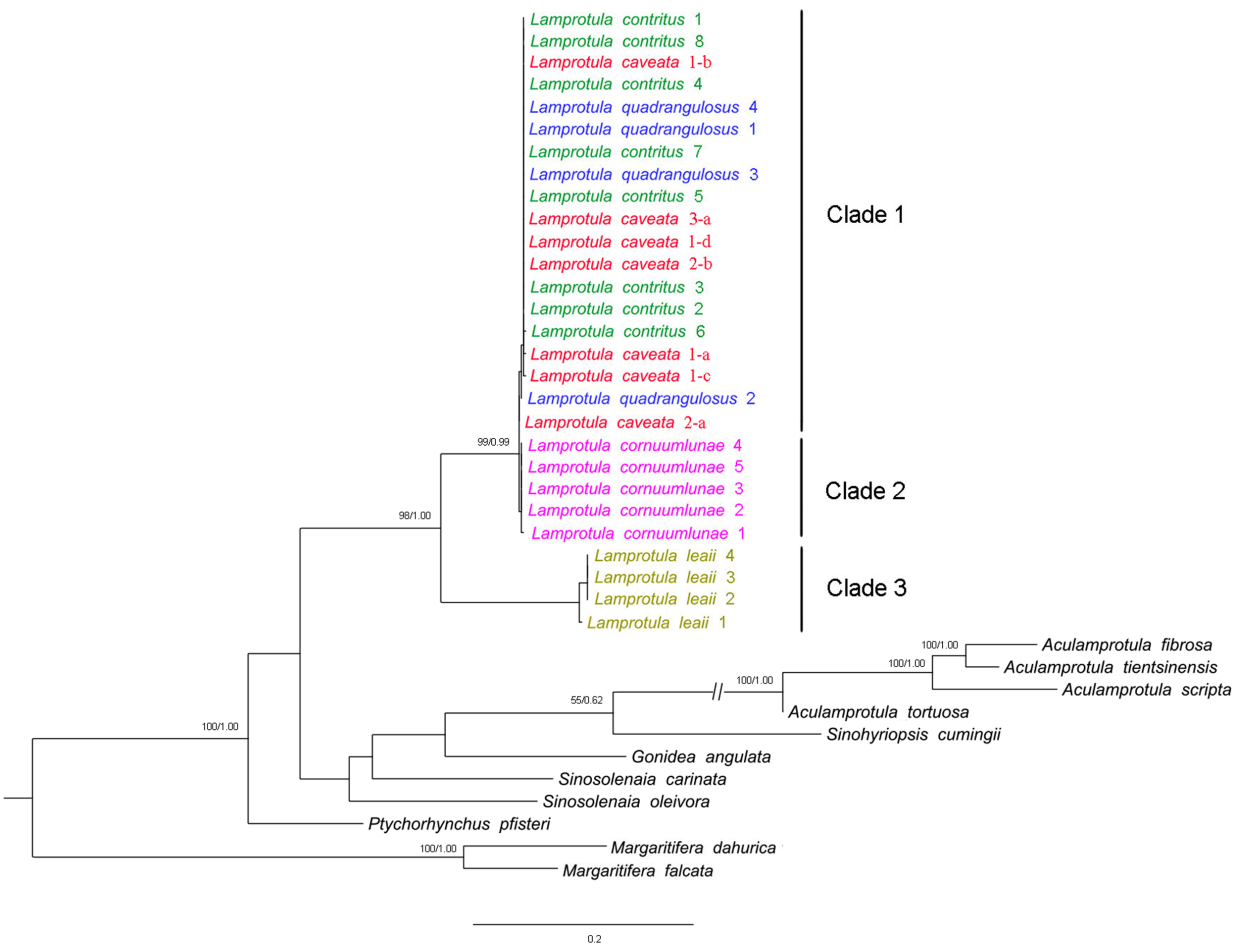
Phylogenetic trees of freshwater mussels obtained by maximum likelihood (ML) and Bayesian inference (BI) analyses based on COI dataset. Support values above the branches are bootstrap support/posterior probabilities. Support value below 50 and 0.5 is not shown. Arabic numerals and letters in the figure correspond to specimen numbers in Figures 2, 3, 4

Mitochondrial COI sequences (DNA barcoding) have been widely used for species delimitation of freshwater mussels based on genetic distance and the criteria of monophyly (Elderkin et al., 2016; Lopes‐Lima et al., 2019; Smith et al., 2018). We extracted the total genomic DNA from dissected somatic tissues using TIANamp Marine Animals DNA Kit (Tiangen Biotech, Beijing, China) according to the manufacturer's instructions. Polymerase chain reaction (PCR) primers for the COI gene regions were LCO1490 (5’‐GGTCAACAAATCATAAAGATATTGG‐3′) and HCO2198 (5’‐TAAACTTCAGGGTGACCAAAAAATCA‐3′). PCR conditions and processes were as follows: 94°C for 5 min; 35 cycles of 94°C for 1 min, 50°C for 1 min, 72°C for 1 min, and a final extension of 72°C for 10 min. Amplified PCR products were purified and sequenced by Sangon Biotech (Shanghai). As a result, a total of 28 COI sequences of *Lamprotula* were used for molecular analysis, including 13 sequences we have previously published (Wu et al., 2020). These *Lamprotula* data were combined with sequences from six species in the subfamily Gonideinae, four species in the subfamily Unioninae, and two species in the family Margaritiferidae (for use as outgroups) obtained from GenBank to complete the dataset (Figure 5; Table S1). All samples and GenBank accession numbers are shown in Table S1.

### 2.3 Phylogenetic analysis and species delimitation

To test the presence of the stop codon and sequencing errors, COI nucleotide sequences were translated to amino acid sequences using MEGA5 (Tamura et al., 2011) and aligned based on the amino acid sequences using the program MUSCLE (Edgar, 2004) with the default setting. The aligned nucleotide sequences (522 bp) were used for the following analyses. We calculated and compared inter‐ and intraspecific distances with MEGA 5.0 using the uncorrected *p*‐distance. Standard error was assessed using 1000 bootstrap replicates (Minh et al., 2013). We generated Bayesian inference (BI) using MrBayes Version 2.01 (Ronquist et al., 2012). Four chains were run simultaneously for 10 million generations, and trees were sampled every 1000 generations. The first 25% of these trees were discarded as burn‐in when computing the consensus tree (50% majority rule). Sufficient mixing of the chains was considered to have been reached when the average standard deviation of split frequencies was below 0.01. Additionally, IQ‐TREE web server (Trifinopoulos et al., 2016) was run for maximum likelihood (ML) tree reconstruction with 1000 bootstrap replicates.

We used two methods for molecular species delimitation: Automatic Barcode Gap Detection (ABGD) and Species Tree And Classification Estimation, Yarely (STACEY). ABGD analyses (Puillandre et al., 2012) were performed at the web server (http://wwwabi.snv.jussieu.fr/public/abgd/) using the default value of relative gap width (X = 1.5) and prior intraspecific divergence values (Pmin = 0.001and Pmax = 0.1). Kimura 2‐P (K80) distance model was selected, which accounts for the more frequent nature of transitional substitutions in protein‐coding sequences. STACEY v.1.2.4 (Jones, 2017) was implemented in BEAST 2.0 (Bouckaert et al., 2014); parameter settings were followed Smith et al. (2019): collapseheight = 0.0001, simcutoff = 1.0, and burn‐in 50%.

### 2.4 Six‐gene data generation, fossil calibrations, and divergence time estimation

To further understand the phylogenetic relationships and evolutionary pattern in the genus *Lamprotula*, we compiled a comprehensive six‐gene dataset and employed BEAST analysis to produce a calibrated phylogenetic framework. These six loci include the mitochondrial COI, 16S rRNA, ND1 and the nuclear 18S rRNA, 28S rRNA, and Histone 3 (H3) gene fragments and were amplified and sequenced using the same primers from Araujo et al. (2017) and Wu, Chen, et al. (2018).

Based on the above‐mentioned COI sequence amplification, we continued to amplify the other five‐gene markers. However, some specimens were not successfully amplified due to the improper preservation of tissue and DNA. For those specimens that failed to be amplified by all the six genes, we did not concatenate the locus data set for analysis. Information for the *Lamprotula* specimens and outgroup species with the Genbank ID for each locus is shown in Table S2.

The alignment of protein‐coding genes (COI, ND1, and H3) was the same as that for the species delimitation data, whereas non‐protein‐coding genes (16S, 18S, and 28S) were directly aligned based on the nucleotide sequences using MUSCLE. After alignment and trimming, the lengths of COI, ND1, H3, 16S, 18S, and 28S sequences were 585 bp, 561 bp, 243 bp, 264 bp, 1357 bp, and 258 bp, respectively. The six‐gene dataset was concatenated (3268 bp) using SequenceMatrix (Vaidya et al., 2011) for phylogenetic analysis employing 12 data partitions based on genes and codon positions. The best‐fit models of nucleotide substitution under the corrected Akaike Information Criterion were also selected by PartitionFinder v1.1.1 (Lanfear et al., 2012) for each partition. Substitution models assigned to each partition are shown in Table S3.

Phylogenetic analysis was implemented in BEAST ver.1.7.5 (Drummond et al., 2012) with the above‐generated models. The uncorrelated lognormal clock model was selected, and the a priori model of the tree was set as the birth–death speciation process. The MCMC was set to 100 million generations, and the sampling frequency was 10,000. After discarding the first 10% of the samples, the independently duplicated log files and tree files were merged in Logcombiner ver.2.3.0. The combined log file parameters were estimated using Tracer ver. 1.5 to ensure that the effective sample size of each parameter exceeds 200. Finally, the maximum pedigree confidence tree was generated in treeAnnotator ver.1.7.5.

We selected three reliable fossil markers to calibrate species differentiation time. All fossils were selected according to our previous study (Wu et al., 2020): (1) Fossil *Lamprotula hungi*, dated to Eocene/Oligocene boundary based on stratigraphy (Schneider et al., 2012), was assigned to the most recent common ancestor (MRCA) of *Lamprotula leaii* and *L. caveata* (min = 34 Ma, exponential prior, lambda = 9.3) following Bolotov, Kondakov, et al. (2017); (2) the oldest fossil Unionidae was from the lower portion of the Morrison Formation in North America. This portion was dated to 150–155 Ma (Kowallis et al., 1998). Following Graf et al. (2015), the minimum age of Unionidae was set to 152 Ma (exponential prior, lambda = 20). (3) The oldest fossil *Shifangella margaritiferiformis*, dated to the Late Triassic (Liu, 1981). We assigned this fossil to the split between Margaritiferidae and Unionidae (stem age = 230 Ma, exponential prior, lambda = 30) following Huang et al. (2018).

### 2.5 Morphometry

Ninety‐four samples were analyzed for conchological morphometry. We used the electronic vernier caliper to measure shell length (L), shell width (W), and shell height (H) with an accuracy of 0.1 mm. Following Klishko et al. (2018), we performed statistical discriminant analysis for the morphometric characters (W/H, H/L, and W/L) using SPSS Statistics 22. The reliability of discrimination was assessed by Wilk's λ.

## 3 RESULTS

### 3.1 Species delimitation

Genetic distance based on mitochondrial COI showed that the intraspecific genetic distance of five *Lamprotula* morphospecies ranged between 0.000 and 0.004. Interspecific genetic distances between comparisons for *L. quadrangulosa*, *L. contritus*, *L. cornuumlunae*, and *L. caveata* were also very low, ranging from 0.000 to 0.008. The interspecific genetic distance between *L. leaii* and the above *Lamprotula* morphospecies ranged from 0.103 to 0.110 (Table S4).

Both ML and BI analyses produced the exact same topology with only minor difference in support values. Phylogenetic tree revealed that *Lamprotula* spp. formed three clades. Clade 1 was an unresolved monophyletic group, *Lamprotula quadrangulosa*, *Lamprotula contritus*, and *Lamprotula caveata* formed a large polytomy group with very shallow branches. *Lamprotula cornuumlunae* formed monophyletic clade 2 and was sister to clade 1. Monophyletic *Lamprotula leaii* was clade 3 and was sister group to (clade 1 + clade 2) (Figure 5).

The results of molecular species delimitation based on ABGD and STACEY showed that *Lamprotula* spp. identified two MOTUs (molecular operational taxonomic units); the *L. leaii* lineage was one MOTU, and the complex lineage (*L. quadrangulosa*, *L. cornuumlunae*, *L. contritus*, and *L. caveata*) was one MOTU.

### 3.2 Time‐calibrated multilocus phylogenetic analysis

**FIGURE 6:**
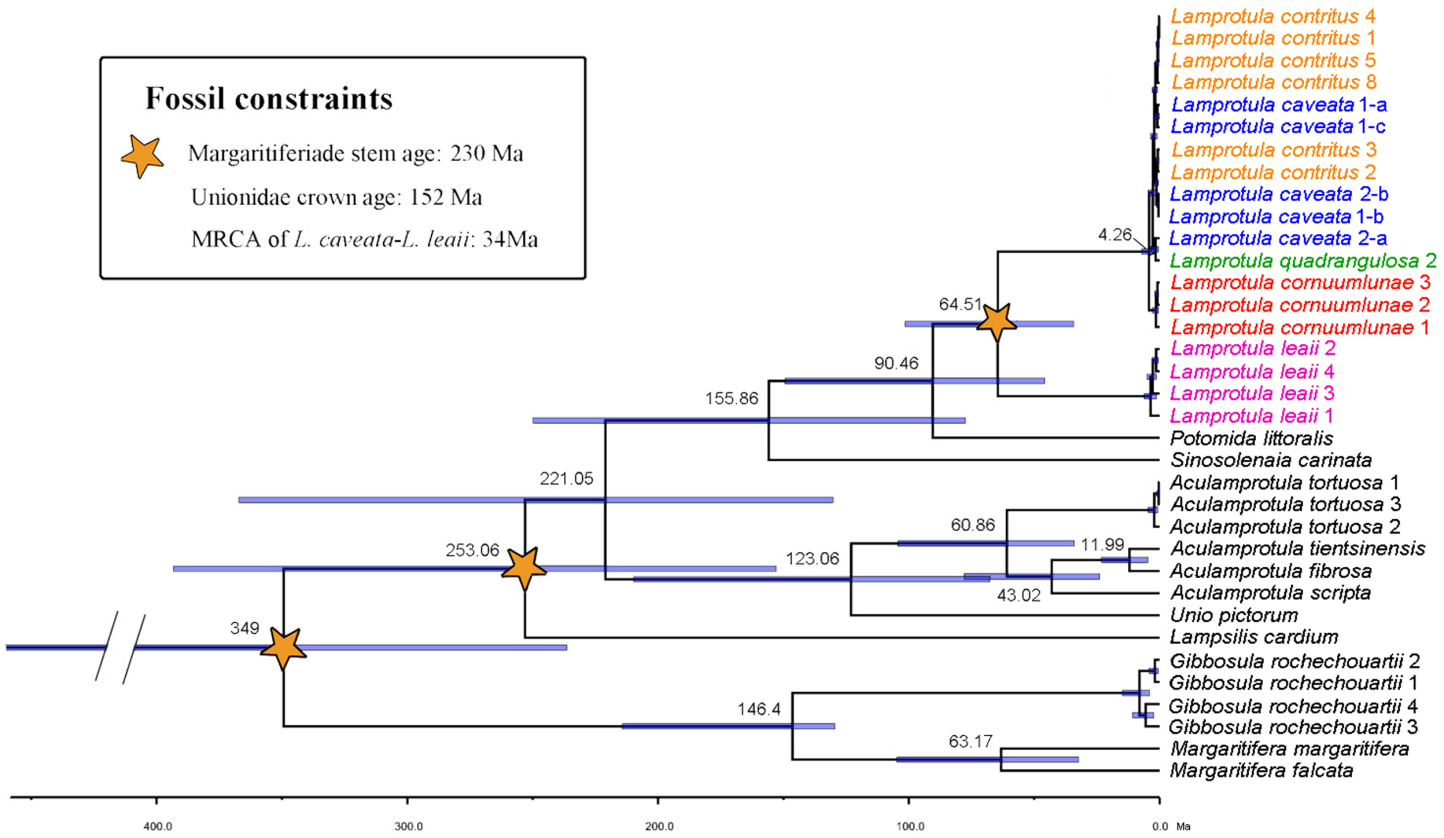
Time‐calibrated six‐locus phylogenetic tree from BEAST analyses. Numbers at nodes are mean age values. Node bars are the 95% highest posterior density (HPD) age estimates. Arabic numerals and letters in the figure correspond to those in Figures 2, 3, 4. Fossils used for calibrations are marked by star signs. MRCA most recent common ancestor, Ma million years ago

The time‐calibrated phylogenetic tree showed that *Lamprotula caveata*, *Lamprotula quadrangulosa*, *Lamprotula cornuumlunae*, and *Lamprotula contritus* formed a monophyletic group and was sister to *Lamprotula leaii*. The differentiation time of *L. leaii* from this larger monophyletic group occurred in Cretaceous (64.51 Ma, 95% HPD [highest posterior density] = 63.22–101.56 Ma). The monophyletic group lineage consisted of two lineages, in which *L. cornuumlunae* formed a basal clade, and *L. contritus*, *L. caveata*, and *L. quadrangulosa* formed a large polytomy group with very shallow branches. *L. cornuumlunae* recently diverged from this group in the Cenozoic Quaternary 4.26 Ma (95% HPD = 1.91–7.22 Ma) (Figure 6).

### 3.3 Morphometry

Shell length ranges per species of *Lamprotula* were as follows: the shell length of *L. caveata* ranged from 43.6 to 90.3 mm; *L. quadrangulosa* was similar in size to the *L. caveata*, and *L. contritus* was measured to be between 25.9 and 47.5 mm in length, which was smaller than the above *Lamprotula* species (Table 1).

**FIGURE 7:**
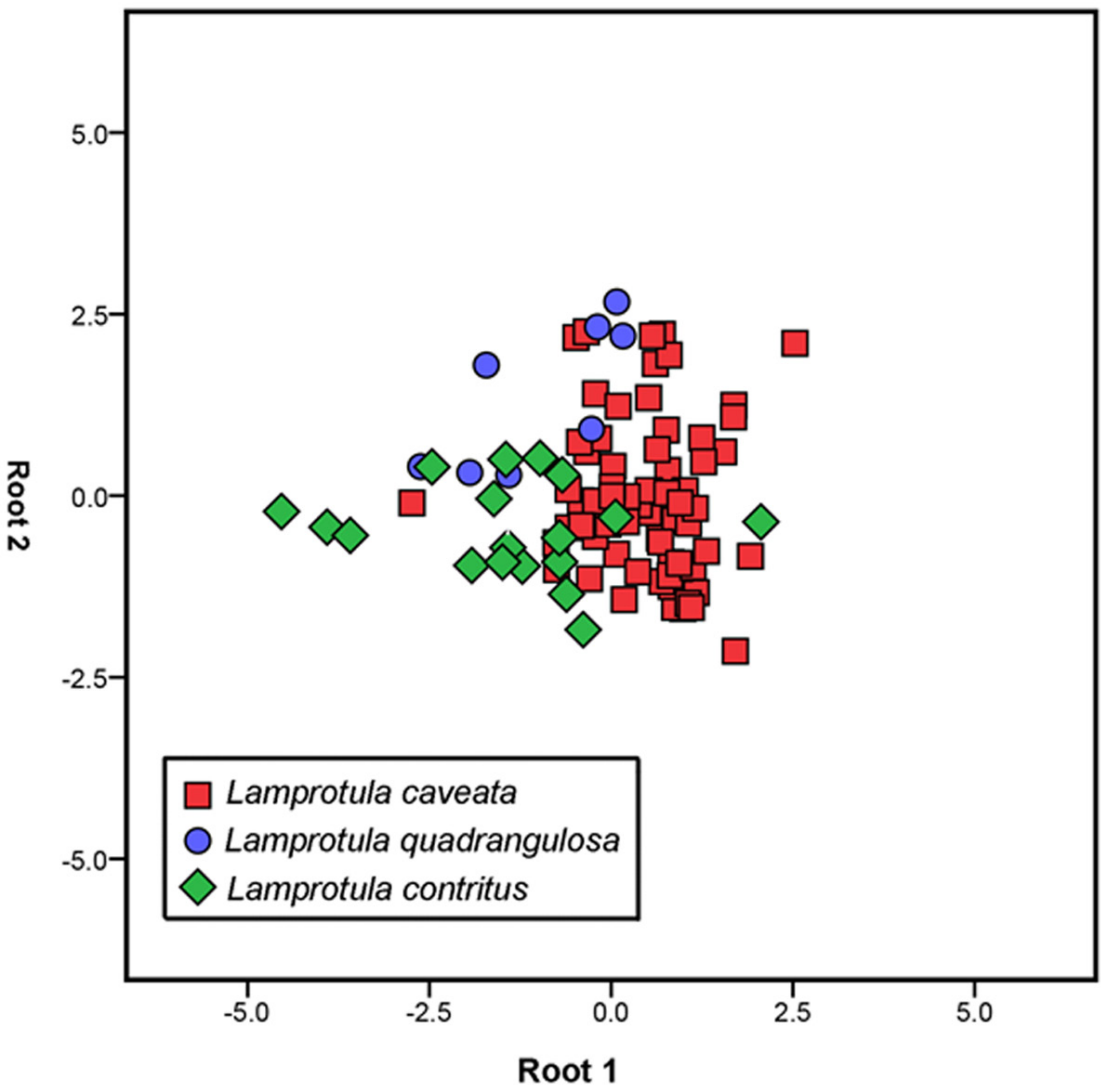
Distribution of *Lamprotula* specimens in the space of the first two discriminant functions based on the morphometric data. Red square indicates *L. caveata* (64 specimens); blue circle indicates *Lamprotula quadrangulosa* (12 specimens); green diamond indicates *L. contritus* (18 specimens)

The scatter plot based on discriminant analyses showed that three *Lamprotula* morphospecies were represented by a single aggregate without clear differentiation into discrete taxa groups (Figure 7). The significance test for discriminant effect showed that the discriminant function was invalid and could not distinguish the three *Lamprotula* morphospecies (λ = 0.497, *n* = 94, *p* < .001).

## 4 DISCUSSION

### 4.1 Synonymization of *L. caveata*

In this study, the phylogenetic trees based on the barcoding and multilocus datasets were generally consistent, revealing that (*L. quadrangulosa* + *L. cornuumlunae* + *L. contritus* + *L. caveata*) formed a monophyletic group, which was sister to *L. leaii*, three taxa (*L. quadrangulosa* + *L. contritus* + *L. caveata*) formed a non‐monophyletic lineage, while *L. cornuumlunae* formed a monophyletic lineage (Figures 5 and 6). Time‐calibrated phylogenetic tree showed that the divergence time of *L. cornuumlunae* was 4.26 Ma, which indicated that divergence time was relatively recent (Figure 6).

Genetic data are frequently used to delimit species, where species status is determined based on genetic distance and the criteria of monophyly (Hebert et al., 2003, 2004; Klishko et al., 2018). Based on ABGD and STACEY for species delimitation, this study seemed to indicate that complex (*L. quadrangulosa* + *L. cornuumlunae* + *L. contritus* + *L. caveata*) was a valid independent species. Interestingly, we observed very shallow branches within (*L. quadrangulosa + L. cornuumlunae + L. contritus + L. caveata*), but *L. cornuumlunae* recovered as monophyletic groups with recent divergence. Species diverged recently will tend to be unrecognized when genetic divergence is the criterion (Hickerson et al., 2006; Knowles & Carstens, 2007; Meier et al., 2006). In the previous study, we have presented the potential problems of relying on genetic sequences for delimiting recently diverged species and demonstrated the validity of *L. cornuumlunae* (Wu et al., 2020). By comparing the conchological characters, Simpson (1914) assert that “the three of Heude's forms (*L. quadrangulosa* + *L. contritus* + *L. caveata*) do not seem to be separable; *L. caveata* is a rudely sculptured form, the other two are smoother” and regarded *L. quadrangulosa* and *L. contritus* as variations of *L. caveata*. Also, shell morphometry of the above three morphospecies was not well separated in this study. Anatomical characters (e.g., marsupium structure, glochidia shape, incurrent aperture, and incurrent aperture) have traditionally been applied to diagnose taxonomic placement among freshwater mussels (Heard, 1974; Heard & Guckert, 1971; Ortmann, 1912). *L. caveata*, *L. cornuumlunae*, and *L. leaii* are indistinguishable in marsupium and glochidia morphology, which were tetragenous brooders of the non‐hooked glochidia (Wu, 1998; Wu, Liu, et al., 2018; Xu et al., 2013). By examining the aperture of *L. quadrangulosa*, *L. contritus*, and *L. caveata*, the morphology was consistent with *L. Leaii* (Wu et al., 2021), with mastoid incurrent aperture and smooth excurrent aperture. Anatomical characteristics vary irregularly among unionid species, which were few useful to define *Lamprotula* taxa. Recently, soft anatomy of unionids is also considered undiagnostic at the subfamily level and tribe level (Lopes‐Lima et al., 2017; Wu et al., 2021). In summary, based on the comprehensive multiple‐dataset approach (morphological, morphometric, and molecular analyses), we support that *L. quadrangulosa* and *L. contritus* as synonyms of *L. caveata*, and shell shape in *L. caveata* is highly variable, surface is uneven and covered with nodules, to almost smooth with few or indistinctive nodules.

### 4.2 Phenotypic plasticity of shell form

Freshwater mussels (order Unionoida) show great variability in the shell morphology (Inoue et al., 2014; Ortmann, 1920; Zieritz & Aldridge, 2009), which may arise through two mechanisms. First is phenotypic plasticity in response to specific environments (Via et al., 1995); the alternative mechanism is related to genetic variation. Unionids have a unique life cycle with an obligate parasitic larval stage (glochidia) that is dependent on a host fish (Kat, 2010; Wächtler et al., 2001). High unpredictable habitats of host fish make the diverse habitats for juvenile mussels. Equally, glochidia are brooded in a specialized marsupium formed by the interlamellar spaces (water tubes) of the gills in female mussels (Simpson, 1914). Female eggs can be fertilized by diverse males via inhalant current, resulting in potentially genetically diverse offspring (Kat, 1984).

**FIGURE 8:**
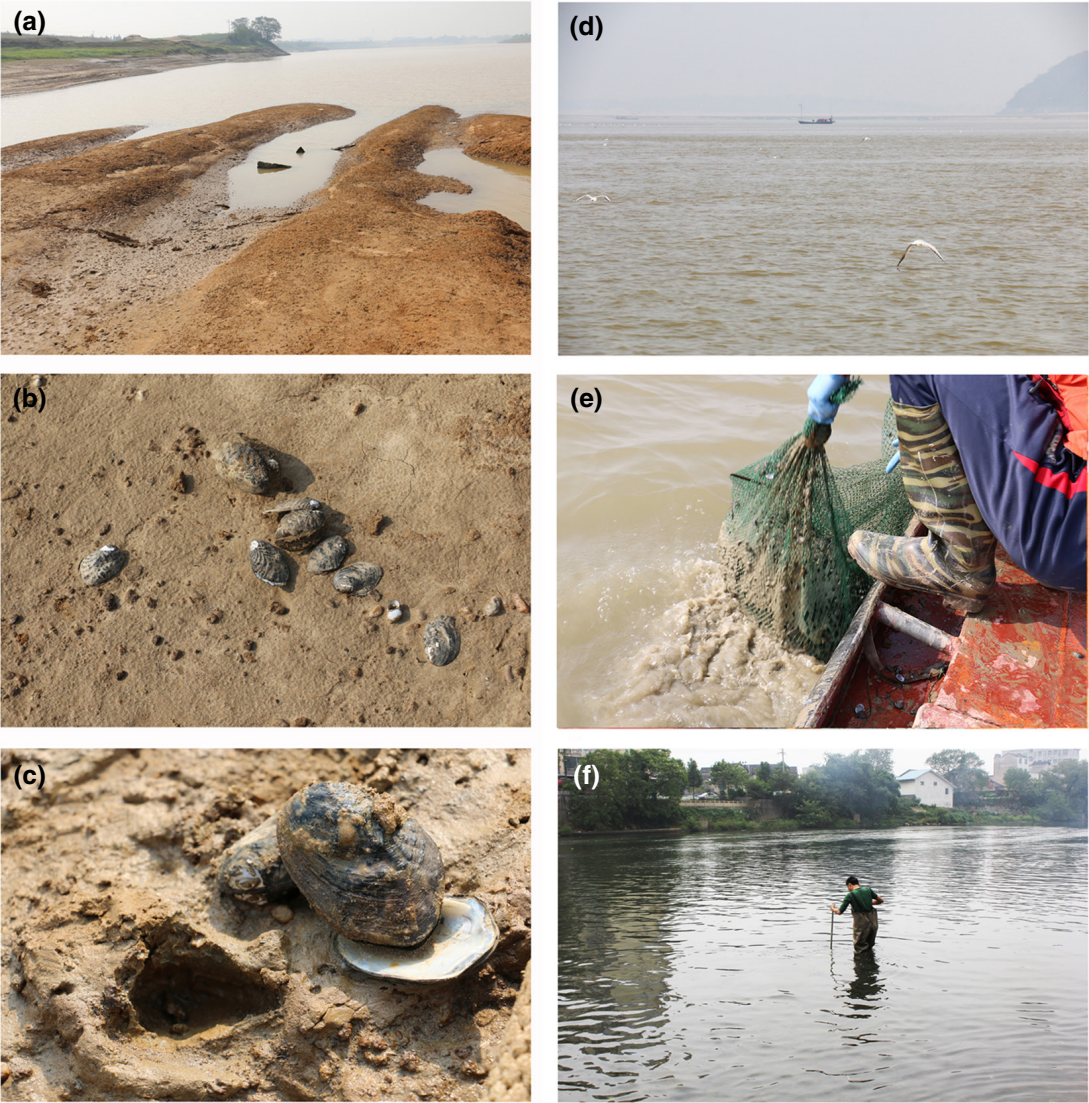
Habitats for *Lamprotula caveata* populations. (a, b and c) the river‐bank soft mud and clay substrate habitat for *L. caveata*. (d and e) the lake—soft mud and clay substrate habitat for *L. caveata* and *L. quadrangulosa* (*L. caveata*). (f) the river—flowed through villages, gravel, and litter substrate habitat for *L. contritus* (*L. caveata*)

The *Lamprotula* in this study were collected from three different habitats (Figure 8). Overall, the present study has shown that individuals sampled from the same habitat were morphologically more similar. Morphospecies *L. contritus* (*L. caveata*) occupies a unique habitat (Figure 8f). Compared with other population habitats, it is located in the residential river with gravel and litter substrate. *L. quadrangulosa* (*L. caveata*) and *L. caveata* occupy homogeneous habitats, but collect different sites. Sediment types and hydrological parameters such as water movement, water quality, and water depth are probably the main factors determining the sculpture, size, and shape of unionids' shells (Klishko et al., 2018; Zieritz et al., 2010). Evidence for phenotypic plasticity of shell morphology has also been found in other mollusks that occupy heterogeneous habitats and have high dispersal potential, which is considered to be an adaptation to a specific environment (Trussell et al., 1993; Vasconcelos et al., 2020). Local population adaptation is capable of driving genetic differentiation (Doebeli & Dieckmann, 2003; Tregenza & Butlin, 1999). However, the haplotypes based on mitochondrial DNA barcodes for *Lamprotula caveata* from different sampling locations did not show clear segregation. Presumably, it is because that gene flow is continuable due to the dispersal of unionids larvae by their host fish in the circulating waters. Extensive gene flow may hinder the formation of specific population genotypes across different habitats. But relatively conserved mitochondrial DNA barcodes lacked adequate resolution to detect genetic differences among populations (Chong et al., 2016). The great level of plasticity and its weak correlation with genetic differentiation was also detected in other species using neutral genetic markers, and other factors such as host fish use may be more important in shaping genetic structure (Geist et al., 2018; Geist & Kuehn, 2005). Whether genotype controls the phenotype for *L. caveata* needs further verification using other molecular markers, for example, microsatellite.

Freshwater mussels are among the most endangered freshwater species worldwide. As China continues to develop economically, additional anthropogenic activities present greater challenges for the conservation of all freshwater organisms, including mussels. This study clarified multiple variational morphologies and provided robust phylogenetics and systematics for the *Lamprotula caveata*, which is of paramount importance to designing effective conservation and management plans, either at local or regional scales.

## AUTHOR CONTRIBUTIONS

**Ruiwen Wu:** Conceptualization (equal); funding acquisition (equal); writing ‐ original draft; writing ‐ review and editing. **Liang Guo:** Investigation (equal). **Chunhua Zhou:** Investigation (equal). **Shan Ouyang:** Resources (equal); software (equal). **Xiaoping Wu:** Funding acquisition (equal); validation (equal). **Xiongjun Liu:** Investigation (equal); resources (equal); software (equal); validation (equal).

## CONFLICT OF INTEREST

None declared.

## Supporting information


Tables S1–S4

## Data Availability

Sequences obtained in this study were uploaded to GenBank, and readers can find GenBank accession numbers in the supplementary tables.
