# Supplementary material for: DNA barcoding, multilocus phylogeny, and morphometry reveal phenotypic plasticity in the Chinese freshwater mussel Lamprotula caveata (Bivalvia: Unionidae)
Source: Ecol Evol. 2022 Jul 13;12(7):e9035. doi: 10.1002/ece3.9035 (PMC9277607; doi:10.1002/ece3.9035)
Supplement: Supplementary file 1 — Tables S1–S4 [file ECE3-12-e9035-s001.pdf]

Appendix A. Supplementary material

Supplementary Table 1 Specimen collection and COI sequence information of the genus *Lamprotula* species and outgroups for the phylogenetic analysis.

Morphospecies	Voucher No.	GenBank no.	Location	Reference
Lamprotula caveata (Heude, 1877) 1-a	FM141017	MG383689	Poyang Lake (116.100544,29.288571) Jiangxi, China	Wu et al., 2020
L. caveata 1-b	FM141018	MG383690	Poyang Lake (116.100544,29.288571) Jiangxi, China	Wu et al., 2020
L. caveata 1-c	FM141019	MG383691	Qinlan Lake (116.193057, 28.559643) Jiangxi, China	Wu et al., 2020
L. caveata 1-d	FM141020	MG383692	Qinlan Lake (116.193057, 28.559643) Jiangxi, China	This study
L. caveata 2-a	FM161021	MG383693	Qinlan Lake (116.193057, 28.559643) Jiangxi, China	Wu et al., 2020
L. caveata 2-b	FM161022	MG383694	Gan River (116.002233,28.876455) Jiangxi, China	Wu et al., 2020
L. caveata 3-a	FM161023	MG383695	Gan River (116.002233,28.876455) Jiangxi, China	This study
Lamprotula quadrangulosa (Heude, 1881) 1	FM161024	MG383696	Qinlan Lake (116.193057, 28.559643) Jiangxi, China	This study
L. quadrangulosa 2	FM161025	MG383697	Qinlan Lake (116.193057, 28.559643) Jiangxi, China	Wu et al., 2020
L. quadrangulosa 3	FM161026	MG383698	Qinlan Lake (116.193057, 28.559643) Jiangxi, China	This study
L. quadrangulosa 4	FM170527	MG383699	Qinlan Lake (116.193057, 28.559643) Jiangxi, China	This study
Lamprotula contritus (Heude, 1883) 1	FM181201	MK682932	Tao River (114.899458, 25.345471) Jiangxi, China	This study
L. contritus 2	FM181202	MK682933	Tao River (114.899458, 25.345471) Jiangxi, China	This study
L. contritus 3	FM181203	MK682934	Tao River (114.899458, 25.345471) Jiangxi, China	This study
L. contritus 4	FM181204	MK682935	Tao River (114.899458, 25.345471) Jiangxi, China	This study
L. contritus 5	FM181205	MK682936	Tao River (114.899458, 25.345471) Jiangxi, China	This study
L. contritus 6	FM170528	MG383700	Tao River (114.899458, 25.345471) Jiangxi, China	This study
L. contritus 7	FM170529	MG383701	Tao River (114.899458, 25.345471) Jiangxi, China	This study

For Review Only

L. contritus 8	FM170530	MG383702	Tao River (114.899458, 25.345471) Jiangxi, China	This study
Lamprotula cornuumlunae (Heude, 1883) 1	FM150401	MK682923	Fu River (116.228282,28.129607) Jiangxi, China	Wu et al., 2020
L. cornuumlunae 2	FM150402	MK682924	Fu River (116.228282,28.129607) Jiangxi, China	Wu et al., 2020
L. cornuumlunae 3	FM150403	MK682925	Fu River (116.228282,28.129607) Jiangxi, China	Wu et al., 2020
L. cornuumlunae 4	FM150404	MK682926	Fu River (116.228282,28.129607) Jiangxi, China	This study
L. cornuumlunae 5	FM150405	MK682927	Fu River (116.228282,28.129607) Jiangxi, China	This study
Lamprotula leaii (Griffith & Pidgeon, 1833) 1	FM180101	MK682928	Poyang Lake (116.228282,28.129607) Jiangxi, China	Wu et al., 2020
L. leaii 2	FM180102	MK682929	Poyang Lake (116.228282,28.129607) Jiangxi, China	Wu et al., 2020
L. leaii 3	FM180103	MK682930	Gan River (115.846214,28.637796) Jiangxi, China	Wu et al., 2020
L. leaii 4	FM180104	MK682931	Gan River (115.846214,28.637796) Jiangxi, China	Wu et al., 2020
Gonidea angulata (Lea, 1838)	-	DQ272373	-	Gustafson & Iwamoto 2005
Ptychorhynchus pfisteri (Heude, 1874)	-	KY067440	-	Zhou et al., 2016
Sinosolenaia carinata (Heude, 1877)	-	NC_023250	-	Huang et al., 2013
Sinosolenaia oleivora (Heude, 1877)	-	NC_022701	-	Huang et al., 2015
Sinohyriopsis cumingii (Lea, 1852)	-	HM347668	-	Unpublished
Aculamprotula fibrosa (Heude, 1877)	-	MG933687	-	Wu et al., 2018a
Aculamprotula scripta (Heude, 1875)	-	MF991456	-	Wu et al., 2018b
Aculamprotula tientsinensis (Crosse & Debeaux, 1863)	-	NC_029210	-	Wu et al., 2016
Aculamprotula tortuosa (Lea, 1865)	-	MG933691	-	Wu et al., 2018a
Margaritifera falcata (Gould, 1850)	-	NC_015476	-	Breton et al., 2011
Margaritifera dahurica (Middendorff, 1850)	-	NC_023942	-	Yang et al., 2015

For Review Only

Reference:

Breton, S., Stewart, D. T., Shepardson, S., Trdan, R. J., Bogan, A. E., Chapman, E. G., Ruminas, A. J., Piontkivska, H., & Hoeh, W. R. (2011). Novel protein genes in animal mtdna: a new sex determination system in freshwater mussels (Bivalvia: Unionoida)? *Molecular Biology and Evolution*, 28, 1645-1659.

Gustafson, R.G., & Iwamoto, E. M. (2005). A DNA-based identification key to Pacific Northwest freshwater mussel glochidia: importance to salmonid and mussel conservation. *Northwest Science*, 79, 233-245.

Huang, X. C., Rong, J., Liu, Y., Zhang, M. H., Wan, Y., Ouyang, S., Zhou, C. H., & Wu, X. P. (2013). The complete maternally and paternally inherited mitochondrial genomes of the endangered freshwater mussel *Solenaia carinatus* (Bivalvia: Unionidae) and implications for Unionidae taxonomy. *Plos One*, 8, e84352.

Huang, X. C., Zhou, C. H., Ouyang, S., & Wu, X. P. (2015). The complete F-type mitochondrial genome of threatened Chinese freshwater mussel *Solenaia oleivora* (Bivalvia: Unionidae: Gonideinae). *Mitochondrial DNA*, 26, 263-264.

Wu, R. W., An, C. T., Wu, X. P., Zhou, C. H., & Ouyang, S. (2016). Complete maternal mitochondrial genome of freshwater mussel *Aculamprotula tientsinensis* (Bivalvia: Unionidae: Unioninae). *Mitochondrial DNA Part A*, 27, 4520-4521.

For Review Only

Wu, R. W., Liu, Y. T., Wang, S., Liu, X. J., Zanatta, D. T., Roe, K. J., Song, X. L., An, C. T., & Wu, X. P. (2018a). Testing the utility of DNA barcodes and a preliminary phylogenetic framework for Chinese freshwater mussels (Bivalvia: Unionidae) from the middle and lower Yangtze River. *Plos One*, 13, e0200956.

Wu, R. W., Wang, S., Liu, Y. T., Liu, X. J., Zhou, C. H., Ouyang, S., & Wu, X. P. (2018b). Characterization and phylogenetic analysis of the complete maternal mitochondrial genome of freshwater mussel *Aculamprotula scripta* (Bivalvia: Unionidae: Unioninae). *Conservation Genetics Resources*, 10, 731-733.

Wu, R. W., Liu, X. J., Ouyang, S. & Wu, X. P. (2020). Comparative Analyses of the Complete Mitochondrial Genomes of Three *Lamprotula* (Bivalvia: Unionidae) Species: Insight into the Shortcomings of Mitochondrial DNA for Recently Diverged Species Delimitation. *Malacologia*, 63(1), 51-66.

Yang, S., Mi, Z., Tao, G., Liu, X., Wei, M., & Wang, H. (2015). The complete mitochondrial genome sequence of *Margaritiana dahurica* Middendorff. *Mitochondrial DNA*, 26, 716-717.

Zhou, C. H., Huang, X. C., Ouyang, S., Ouyang, J. X., & Wu, X. P. (2016). Characterization of the complete maternal mitochondrial genome of *Ptychorhynchus pfisteri* (Bivalvia: Unionidae: Gonideinae). *Conservation Genetics Resources*, 9, 233-235.

For Review Only

For Review Only

Supplementary Table 2 Genbank ID. information of six molecule markers

Family	Subfamily	Morphospecies	COI	ND1	16S	18S	28S rRNA's expansion segment D3	histone H3
Unionidae	Gonideinae	*Lamprotula contritus* 1	MK682932	MK682946	MK683019	MK682994	MK683044	MK682953
		Lamprotula contritus 2	MK682933	MK682947	MK683020	MK682995	MK683045	MK682954
		Lamprotula contritus 3	MK682934	MK682948	MK683021	MK682996	MK683046	MK682955
		Lamprotula contritus 4	MK682935	MK682949	MK683022	MK682997	MK683047	MK682956
		Lamprotula contritus 5	MK682936	MK682950	MK683023	MK682998	MK683048	MK682957
		Lamprotula contritus 8	MG383702	MG386418	MK683024	MK682999	MK683049	MK682958
		Lamprotula caveata 1-a	MG383689	MG386405	MK683006	MK682981	MK683031	MK682962
		Lamprotula caveata 1-b	MG383690	MG386406	MK683007	MK682982	MK683032	MK682963
		Lamprotula caveata 1-c	MG383691	MG386407	MK683008	MK682983	MK683033	MK682964
		Lamprotula caveata 2-a	MG383693	MG386409	MK683009	MK682984	MK683034	MK682965
		Lamprotula caveata 2-b	MG383694	MG386410	MK683010	MK682985	MK683035	MK682966
		Lamprotula quadrangulosa 2	MG383697	MG386413	MK683011	MK682986	MK683036	MK682967
		Lamprotula cornuumlunae 1	MK682923	MK682937	MK683012	MK682987	MK683037	MK682968
		Lamprotula cornuumlunae 2	MK682924	MK682938	MK683013	MK682988	MK683038	MK682969
		Lamprotula cornuumlunae 3	MK682925	MK682939	MK683014	MK682989	MK683039	MK682970
		Lamprotula leaii 1	MK682928	MK682942	MK683015	MK682990	MK683040	MK682971
		Lamprotula leaii 2	MK682929	MK682943	MK683016	MK682991	MK683041	MK682972

For Review Only

		Lamprotula leaii 3	MK682930	MK682944	MK683017	MK682992	MK683042	MK682973
		Lamprotula leaii 4	MK682931	MK682945	MK683018	MK682993	MK683043	MK682974
		Potomida littoralis	KP217871	NC_030073	KP217981	KU763287	KU763330	KU763373
		Sinosolenaia carinata	NC_023250	NC_023250	MK683025	MK683000	MK683050	MK682975
	Unioninae	*Aculamprotula tortuosa* 1	MG933691	MG933752	MK683003	MK682978	MK683028	MK682959
		Aculamprotula tortuosa 2	MG933691	MG933752	MK683004	MK682979	MK683029	MK682960
		Aculamprotula tortuosa 3	MG933691	MG933752	MK683005	MK682980	MK683030	MK682961
		Aculamprotula tientsinensis	MF072504	MG933750	MF072511	MF072525	MF072518	MF072532
		Aculamprotula fibrosa	MG933687	MG933746	MK683001	MK682976	MK683026	MK682951
		Aculamprotula scripta	MF991456	MF991456	MK683002	MK682977	MK683027	MK682952
		Unio pictorum	KC429109	NC_015310	KC429266	KC429349	KC429447	KC429186
	Ambleminae	*Lampsilis cardium*	KX713472	FJ601358	KX713226	KX713305	KX713394	KX713547
Margaritiferidae	Gibbosulinae	*Gibbosula rochechouartii* 1	MF072499	MK681924	MF072506	MF072520	MF072513	MF072527
		Gibbosula rochechouartii 2	MF072498	MK681925	MF072505	MF072519	MF072512	MF072526
		Gibbosula rochechouartii 3	MF072501	MK681926	MF072508	MF072522	MF072515	MF072529
		Gibbosula rochechouartii 4	MF072500	MK681927	MF072507	MF072521	MF072514	MF072528
	Margaritiferinae	*Margaritifera falcata*	AY579128	NC_015476	AY579085	AY579101	AY579117	AY579141
		Margaritifera margaritifera	KU763227	NC_021638	KU763196	KU763258	KU763301	KU763345

For Review Only

Supplementary Table 3 Partitioning strategies from PartitionFinder for six-molecular marker dataset.

Partition Part	Model	Length	Gene
1	GTR+I+G+X	451	ND1_pos1, 16S
2	GTR+I+G+X	1777	28S, 18S, H3_pos1, H3_pos2
3	GTR+I+G+X	658	COI_pos2, ND1_pos2, H3_pos3, COI_pos1
4	TRN+G+X	195	COI_pos3
5	HKY+G+X	187	ND1_pos3

For Review Only

Supplementary Table 4 Intraspecific and interspecific genetic distances based on mitochondrial COI gene for *Lamprotula* species.

Morphospecies	Intraspecific distances	Interspecific distances									
Lamprotula caveata 1-a											
Lamprotula caveata 1-b		0.002									
Lamprotula caveata 1-c		0.004	0.002								
Lamprotula caveata 1-d	0.004	0.002	0.000	0.002							
Lamprotula caveata 2-a		0.006	0.004	0.006	0.004						
Lamprotula caveata 2-b		0.002	0.000	0.002	0.000	0.004					
Lamprotula caveata 3-a		0.002	0.000	0.002	0.000	0.004	0.000				
Lamprotula quadrangulosa	0.001	0.002	0.002	0.000	0.000	0.002	0.000	0.000			
Lamprotula cornuumlunae	0.001	0.008	0.006	0.008	0.006	0.003	0.006	0.006	0.006		
Lamprotula contritus	0.000	0.002	0.000	0.002	0.000	0.004	0.000	0.000	0.001	0.006	
Lamprotula leaii	0.004	0.110	0.108	0.110	0.108	0.103	0.108	0.108	0.107	0.106	0.107

For Review Only
